# Community Social Capital, Built Environment, and Income-Based Inequality in Depressive Symptoms Among Older People in Japan: An Ecological Study From the JAGES Project

**DOI:** 10.2188/jea.JE20160216

**Published:** 2018-03-05

**Authors:** Maho Haseda, Naoki Kondo, Toyo Ashida, Yukako Tani, Daisuke Takagi, Katsunori Kondo

**Affiliations:** 1 Department of Health and Social Behavior and Department of Health Sociology and Health Education, The University of Tokyo, Tokyo, Japan; 2 Graduate School of Economics, The University of Tokyo, Tokyo, Japan; 3 Department of Global Health Promotion, Tokyo Medical and Dental University, Tokyo, Japan; 4 Research Fellow of the Japan Society for the Promotion of Science, Tokyo, Japan; 5 Center for Preventive Medical Sciences, Chiba University, Chiba, Japan; 6 Center for Gerontology and Social Science, National Center for Geriatrics and Gerontology, Aichi, Japan

**Keywords:** social capital, health inequality, depression, aged, Japan

## Abstract

**Background:**

Although reducing socioeconomic inequalities in depression is necessary, their associated factors have rarely been studied. This study aimed to screen the potential contextual factors associated with income-based inequality in older adults’ depression.

**Methods:**

Using data from the Japan Gerontological Evaluation Study (JAGES) of 2013, we conducted an ecological study covering 77 communities in Japan. Our measures of socioeconomic inequalities in depression were the slope index of inequalities (SII) and the relative index of inequalities (RII) of the prevalence of depressive symptoms across three income levels. We categorized available community-level factors, including socio-demographic factors, social participation, social relationships, subjective changes in the residential area, and the built environment. These indicators were aggregated from individual responses of 51,962 and 52,958 physically independent men and women, respectively, aged 65 years or more. We performed multiple linear regression analyses to explore factors with statistical significance of a two-tailed *P*-value less than 0.05.

**Results:**

Factors associated with shallower gradients in depression for men included higher participation in local activities and reception or provision of social support, which did not show significant association among women. Perceived increases in unemployment and economic inequalities were positively associated with larger inequalities in both genders (*P* < 0.05). The built environment did not indicate any significant association.

**Conclusions:**

A community environment fostering social activities and relationships might be associated with smaller income-based inequalities in depression. There is a need for more deterministic studies for planning of effective community interventions to address socioeconomic inequalities in depression.

## INTRODUCTION

Depression among older people is a known risk factor for suicide,^1^ a decline in daily living activities,^2^ poor quality of life,^3^ and poor disease prognosis.^4^ Studies from Japan and many parts of the world have revealed that depression is more prevalent among low-income and other socioeconomically vulnerable groups.^5^^,^^6^ Depression is associated with neighborhood contexts. Community-level factors potentially causing depressive symptoms include income inequality,^7^ neighborhood crime, unsafe traffic, and unwillingness to support each other.^8^

However, these data do not explain socioeconomic *inequalities* in the prevalence of depressive symptoms. To the best of our knowledge, there are no studies examining the association between community-level factors and socioeconomic inequalities in depressive symptoms. Identifying community-level factors explaining the levels of inequalities in depression is essential for the development of strategies to reduce the inequalities.

We hypothesized two possible reasons why neighborhood factors affect socioeconomic inequalities in mental illness. First, people with a lower income have less access to resources to maintain their health, which is presumably more likely in resource-scarce community settings. For example, they may be likely to experience delays in care and unmet medical needs, and to have fewer opportunities for social participation, due to unequally distributed services.^9^^,^^10^ Second, community social cohesion may be more beneficial for socioeconomically vulnerable populations, by facilitating collective action, mutual support, and informal social control, potentially resulting in more effective allocation of resources that are necessary for maintaining good mental health.^11^

Therefore, using large-scale data from a survey on Japanese older adults, we performed an ecological study to explore the community factors associated with the amplitude of income-based inequalities in depressive symptoms among older adults. We aimed to identify possible/hypothetical factors, to be followed up with a more detailed study on the community environment and depression-related inequalities.

## METHODS

### Data

We used data from the 2013 survey of the Japan Gerontological Evaluation Study (JAGES). In the study, we conducted a mail-in survey of individuals aged 65 years or older, who were not receiving any benefits from public long-term care insurance. Participants were randomly selected from 16 large municipalities through multistage sampling and, in addition, all eligible individuals were selected from 14 small municipalities. We distributed 193,694 self-reported questionnaires and received 137,736 (response rate = 71.1%). After excluding the data of subjects whose information on municipality (*n* = 561) or household income (*n* = 26,531) were missing, we used the data of 51,962 men and 52,958 women. Subjects receiving public financial assistance have access to medical care without out-of-pocket payment, which may result in a specific pattern of depression prevalence among them. However, we did not exclude such subjects because we confirmed that their prevalence of depressive symptoms was not lower than that of the lower-income group overall (23.6% and 11.4%, respectively). Among the 30 municipalities, four municipalities with large populations (ie, metropolitan cities) were divided into smaller administrative wards. This created a total of 77 communities in our data.

We extracted information on population density and the proportion of the population aged 65 years or more from the database of the Statistics Bureau in Japan.^12^

### Measurement

#### Dependent variables

For the dependent variables of this community-level ecological study, we calculated multiple measures representing within-district income-based inequalities in the prevalence of depressive symptoms among men and women. We assessed depressive symptoms using the Japanese short version of the Geriatric Depression Scale (GDS-15) developed for self-administrative surveys.^13^^,^^14^ We used a cut-off score of 10, with scores ≥10 indicating severe depressive symptoms, which would account for about 11.3% of community-dwelling Japanese elderly subjects.^3^^,^^15^ A validation study has shown that a cutoff score of 9/10 on the GDS-15 for community dwelling older adults in Iran had a sensitivity of 0.82, specificity of 0.86, positive predictive value (PPV) of 0.41, and negative predictive value (NPV) of 0.98 for major depressive disorder diagnosis based on the Composite International Diagnostic Interview (CIDI).^16^ We calculated the prevalence of severe depression by communities, gender, and income tertile, with adjustment for age distribution using direct standardization. As for income, we asked respondents’ annual household pre-tax income. We divided the household income by the square root of the number of family members and converted to equalized household income to facilitate consideration of household composition.

#### Independent variables

For the independent variables, we selected basic socio-demographic factors and modifiable local community factors, including social participation, social relationships, perceived changes in residential areas, and the built environment in each community. These factors were selected based on the JAGES Health Equity and Response Tool (JAGES HEART).^17^ The JAGES HEART was developed for benchmarking health statuses at the community level among older populations.^18^

Basic socio-demographic factors include the percentages of people with low income (less than 2 million yen for equivalized household income, following a previous report by the JAGES group^5^) and low education levels (less than 9 years); the proportions of the population aged 65 years or more, of older people who live alone, and of people reporting a history of diseases previously reported to be associated with depression (stroke, heart disease, diabetes mellitus, cancer, dementia, and Parkinson’s disease); and population density by inhabited area.

As for social relationships and social participation, we chose multiple indicators articulating these concepts. For structural social capital, we used the proportion of subjects who reported previous participation in the following local activities several times per year or more: “volunteers’ group”, “sports group or club”, “leisure activity club”, “senior-citizen club”, “neighborhood association or residents’ association”, “study or cultural group”, “nursing care prevention or health-building activities”, “activities to teach skills or pass on experience to others”, “local events”, “activities to support older people requiring protection”, “activities to support older people requiring nursing care”, “activities to support parents raising children”, and “neighborhood beautification activities.” For social relationships, we used the proportion of people who reported having friends to meet, having someone to receive emotional or instrumental social support from or to provide such support for, trusting people in the area in general, thinking that people in the area try to help each other, being attached to the residential area, and co-operating in daily life with neighbors. When selecting these variables, we referred to the definition of community social capital as “resources that are accessed by individuals as a result of their membership of a network or a group.”^11^ We used the Gini coefficient to evaluate within-community income inequality.^19^ The precise definitions of each variable are described in eTable 1.

#### Health inequality measures

To monitor health inequalities, a study has recommended use of not only simple measures of differences and ratios of health indicators across socioeconomic groups, but also more sophisticated indicators. Among those alternative indicators, the slope index of inequality (SII) and the relative index of inequality (RII) are recommended, as they have advantages in precision and comparability.^20^ Thus, for this study we used the SII and the RII, as well as differences and ratios of depressive symptoms’ prevalence. The SII and the RII require the assumption of linear and monotone associations between income levels and depressive symptoms. To calculate the SII, each social group is first ordered from lowest to highest on the x-axis with the cumulative distribution of the population. Then, health status (y_i_), which is the mean score of each group, is plotted above the midpoint of its range in the cumulative distribution in the population of each socioeconomic category (x_i_). The SII is the regression coefficient (β_1_) for y_i_ (= β_0_ + β_1_x_i_).^21^ The RII is a relative measure derived from the SII divided by the mean of the health parameter. The SII can be interpreted as the estimated absolute difference in health status across the entire distribution of social groups. The advantages of these indices include accounting for changes in the population distributions in the social groups over time and the utilization of information across entire social groups. We used Health Disparity Calculator version 1.2.4 (National Cancer Institute, Rockville, MD, USA).^22^

### Statistical analysis

We performed linear regression analyses and sought out factors with the statistical significance of two-tailed *P*-values less than 0.05. Considering that community factors might affect men and women differently, as reported in previous studies,^23^^,^^24^ we performed stratified analyses by gender. We adjusted for basic community compositional characteristics that potentially confounded to our analyses: the proportions of the population aged 65 years or more, the proportions having a low income (less than 2 million JPY for equivalized household income), the proportions having a low education level (9 years or less), the proportions of older people living alone, the proportions having a history of diseases associated with depression, and population density. We evaluated variance inflation factors (VIF) to prevent strong multicollinearity.

To confirm robustness, we performed sensitivity analyses, using an alternative cut-off value (5 points, representing mild depressive symptoms) for the GDS-15.^25^ We used Stata version 14.1 for these statistical analysis (Stata Corp., College Station, TX, USA).

### Ethics approval

This study was approved by the Ethics Committee in Research of Human Subjects at Nihon Fukushi University (13-14), Chiba University Faculty of Medicine (No. 1777), and the Ethics Committee at the University of Tokyo Faculty of Medicine (10555).

## RESULTS

**Figure 1.**
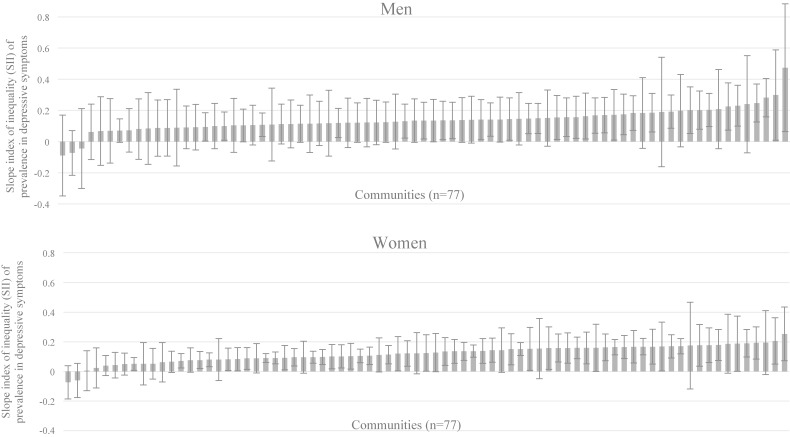
Cross-municipality inequalities of prevalence in depressive symptoms by gender: Slope Index of Inequality (SII) . Error bars indicate 95% Confidence Intervals.

Overall, the prevalence of depressive symptoms was high among low-income participants. Age-adjusted prevalence of depressive symptoms in the total population was 12.2% in men and 10.7% in women, 7.2% in men and 5.5% in women, and 3.2% in men and 3.0% in women in the low-, middle-, and high-income groups, respectively (Table 1). We found wide between-community variations in within-community inequality with regard to depressive symptoms (Figure 1). We also identified large variations in the variables representing community characteristics (Table 2).

**Table 1.** Income-based inequality statistics and indices in depressive symptoms for older Japanese men (*n* = 51,962) and women (*n* = 52,958)

	Income Tertile	Mean (SD)	Rate Difference	Rate Ratio	Slope Index of Inequality (SII)	Relative Index of Inequality (RII)
Men	T1 (low)	12.2% (4.5%)				
	T2 (middle)	7.2% (3.6%)				
	T3 (high)	3.2% (2.1%)	9.0%	3.81	0.14	2.03

Women	T1 (low)	10.7% (3.7%)				
	T2 (middle)	5.5% (2.7%)				
	T3 (high)	3.0% (1.7%)	7.7%	3.61	0.12	1.83

SD, standard deviation.

**Table 2.** Age-adjusted descriptive statistics of community factors

	Mean	(SD)
**Socio-demographic factors**		
% Income <200 JPY	49.8%	(9.4%)
% Education <9 years	36.9%	(13.3%)
% Age ≥65 years^a^	22.6%	(4.9%)
% Living alone	16.1%	(5.6%)
% Have comorbidities^b^	26.1%	(1.9%)
Population density, /km^2 a^	5,351.7	(4,141.2)
**Social participation**		
Volunteer group	20.6%	(4.1%)
Sports club/group	30.3%	(5.8%)
Leisure activity club	42.4%	(5.1%)
Senior citizens’ club	18.6%	(9.8%)
Neighborhood association	38.0%	(9.7%)
Cultural group	15.4%	(2.9%)
Health-promoting activities	13.8%	(3.7%)
Activities entailing passing on experience to others	11.6%	(2.0%)
Local events	30.0%	(9.7%)
Supporting older people requiring protection	8.1%	(3.5%)
Supporting older people requiring long-term care	6.6%	(2.2%)
Supporting parents raising children	6.4%	(1.3%)
Local beautification activities	26.7%	(9.3%)
**Social relationships**		
Having friends	91.0%	(2.3%)
Receiving emotional social support	94.2%	(1.3%)
Providing emotional social support	92.5%	(1.3%)
Receiving instrumental social support	94.4%	(2.2%)
Providing instrumental social support	80.1%	(2.6%)
Trusting people in the area (very much or moderately)	68.6%	(4.8%)
Practicing reciprocity as a norm (very much or moderately)	52.3%	(6.2%)
Having a sense of attachment to the neighborhood (very much or moderately)	79.1%	(3.0%)
Co-operating with neighbors (very much or moderately)	16.2%	(8.9%)
**Perceived changes in the area**		
Revitalization of the local economy	4.9%	(2.1%)
Depression of the local economy	11.6%	(4.7%)
Deterioration of security	7.3%	(3.7%)
More newcomers	17.2%	(6.3%)
Decline in local festivities	13.1%	(5.5%)
Increase in unemployment	2.6%	(1.1%)
Increase in poverty	2.4%	(1.1%)
Improvement of administrative services	2.5%	(1.4%)
Deterioration of administrative services	5.6%	(2.7%)
Widening income inequality	5.3%	(1.4%)
More local activities	5.8%	(2.5%)
Fewer local activities	13.4%	(3.7%)
**Built environment**		
Graffiti or garbage	25.5%	(6.3%)
Exercise environment	79.1%	(10.9%)
Hills or steps	44.3%	(19.4%)
Risk of traffic accidents	62.5%	(6.5%)
Fascinating views	47.2%	(12.2%)
Shops selling fresh foods	78.0%	(10.8%)
Dangerous places to walk in alone at night	59.5%	(6.2%)
Places to feel free to drop in	41.1%	(6.2%)

SD, standard deviation.

All factors were adjusted for age.

^a^Data from the Statistics Bureau in Japan.

^b^Stroke, heart disease, diabetes mellitus, cancer, dementia, and Parkinson’s disease.

Among men, communities with high social participation in various activities were inversely associated with inequalities in the prevalence of depressive symptoms (Table 3). Adjustment for covariates attenuated these associations, but participation in sports clubs/groups, senior citizens’ clubs, and cultural activities remained significantly associated with the SII. Participation in sports clubs was also significantly associated with the RII. Similarly, most social relationship indicators were inversely associated with the SII and the RII. Specifically, social support indices—both their provision and reception—were strongly associated with the SII, even after adjusting for covariates. For example, the adjusted standardized coefficient (β) for the percentage of reception of instrumental social support was −0.55 (*P* = 0.008). Compared to this, the associations of perceived changes in community characteristics with the SII and the RII were weaker, with a few exceptions. The percentage of participants perceiving increased poverty was positively associated with high SII (adjusted β = 0.24, *P* = 0.06); this was also true for increased unemployment (adjusted β = 0.31, *P* = 0.03) and widening income inequality (adjusted β = 0.23, *P* = 0.09).

**Table 3.** Association between income-based health inequality indices (slope index of inequality [SII] and relative index of inequality [RII]) in depressive symptoms by a unit increase of 1 standard deviation in measures of community characteristics among older Japanese men (*n* = 51,962): multiple regression results

	SII				RII			
	
	Crude		Adjusted^a^		Crude		Adjusted^a^	
			
	β	*P*	β	*P*	β	*P*	β	*P*
**Socio-demographic factors**								
% Income <200 JPY	−0.16	0.165			−0.43^*^	<0.001		
% Education <9 years	0.06	0.601			0.16	0.167		
% Age ≥65 years^b^	0.14	0.231			−0.15	0.185		
% Living alone	0.23^*^	0.041			0.00	0.974		
% Have comorbidities^c^	0.28^*^	0.013			0.32^*^	0.005		
Population density, /km^2 b^	0.01	0.948			−0.19	0.098		
Gini coefficient	0.23^*^	0.042	0.08	0.543	0.03	0.77	0.08	0.541
**Social participation**								
Volunteer group	−0.29^*^	0.011	−0.18	0.205	−0.38^*^	0.001	−0.21	0.125
Sports club/group	−0.26^*^	0.021	−0.27^*^	0.041	0.05	0.638	−0.41^*^	0.001
Leisure activity club	−0.23^*^	0.047	−0.24	0.105	0.11	0.327	−0.22	0.121
Senior citizens’ club	−0.29^*^	0.012	−0.37^*^	0.029	−0.33^*^	0.003	0.05	0.756
Neighborhood association	−0.25^*^	0.029	−0.19	0.208	−0.31^*^	0.006	−0.14	0.340
Cultural group	−0.21	0.073	−0.24^*^	0.040	0.08	0.493	−0.08	0.496
Health-promoting activities	−0.23^*^	0.042	−0.21	0.117	−0.23^*^	0.040	0.03	0.812
Activities entailing passing on experience to others	−0.16	0.176	−0.08	0.444	−0.04	0.699	−0.19	0.080
Local events	−0.30^*^	0.009	−0.19	0.217	−0.42^*^	<0.001	−0.13	0.396
Supporting older people requiring protection	−0.24^*^	0.034	−0.29	0.082	−0.43^*^	<0.001	−0.03	0.875
Supporting older people requiring long-term care	−0.26^*^	0.023	−0.28	0.051	−0.43^*^	<0.001	−0.07	0.631
Supporting parents raising children	−0.14	0.220	−0.03	0.845	−0.19	0.099	−0.10	0.460
Local beautification activities	−0.24^*^	0.036	−0.20	0.216	−0.28^*^	0.012	−0.23	0.149
**Social relationships**								
Having friends	−0.30^*^	0.007	−0.27	0.076	−0.25^*^	0.031	0.06	0.715
Receiving emotional social support	−0.38^*^	0.001	−0.29^*^	0.028	−0.14	0.226	−0.14	0.282
Providing emotional social support	−0.33^*^	0.003	−0.19	0.140	−0.17	0.145	0.05	0.714
Receiving instrumental social support	−0.35^*^	<0.001	−0.55^*^	0.008	−0.05	0.680	−0.02	0.925
Providing instrumental social support	−0.37^*^	<0.001	−0.32^*^	0.045	−0.16	0.157	0.03	0.871
Trusting people in the area (very much or moderately)	−0.28^*^	0.013	−0.03	0.823	−0.10	0.395	−0.15	0.313
Practicing reciprocity as a norm (very much or moderately)	−0.33^*^	0.004	−0.13	0.438	−0.30^*^	0.008	−0.32	0.057
Having a sense of attachment to the neighborhood (very much or moderately)	−0.23^*^	0.043	−0.03	0.824	−0.05	0.649	0.01	0.924
Co-operating with neighbors (very much or moderately)	−0.19	0.090	−0.04	0.849	−0.35^*^	0.002	0.26	0.232
**Perceived changes in the area**								
Revitalization of the local economy	−0.10	0.394	−0.14	0.211	0.03	0.807	0.11	0.303
Depression of the local economy	0.05	0.659	0.15	0.286	−0.20	0.077	0.13	0.350
Deterioration of security	0.11	0.326	−0.02	0.897	0.13	0.273	0.15	0.224
More newcomers	0.00	0.985	−0.23	0.135	0.18	0.109	−0.29	0.050
Decline in local festivities	−0.11	0.358	−0.04	0.734	−0.27^*^	0.017	0.21	0.088
Increase in unemployment	0.10	0.391	0.31^*^	0.030	−0.20	0.080	−0.18	0.207
Increase in poverty	0.24^*^	0.033	0.24	0.059	0.06	0.620	−0.13	0.316
Improvement of administrative services	−0.12	0.291	−0.09	0.450	0.11	0.320	−0.13	0.229
Deterioration of administrative services	−0.08	0.499	−0.02	0.864	−0.23^*^	0.046	0.13	0.368
Widening income inequality	0.14	0.209	0.23	0.090	0.07	0.549	−0.03	0.808
More local activities	−0.22	0.060	−0.10	0.438	−0.08	0.467	−0.16	0.192
Fewer local activities	−0.06	0.619	−0.01	0.940	−0.26^*^	0.024	0.27	0.058
**Built environment**								
Graffiti or garbage	0.19	0.091	−0.01	0.935	0.03	0.801	0.05	0.714
Exercise environment	0.12	0.291	0.12	0.541	0.30^*^	0.009	−0.23	0.213
Hills or steps	−0.10	0.402	0.00	0.973	−0.04	0.706	−0.24	0.058
Risk of traffic accidents	0.16	0.161	0.03	0.793	0.17	0.140	0.26	0.035
Fascinating views	0.13	0.253	0.11	0.378	0.16	0.175	−0.13	0.287
Shops selling fresh foods	0.22	0.057	0.31	0.123	0.36^*^	0.001	−0.21	0.293
Dangerous places to walk in alone at night	−0.10	0.370	−0.07	0.535	−0.10	0.390	−0.02	0.883
Places to feel free to drop in	−0.13	0.244	0.01	0.956	−0.19	0.107	0.02	0.858

All factors were adjusted for age.

^*^P < 0.05.

^a^Adjusted for the proportion of the population aged 65 years or more, the proportion of people who live alone, the proportion of people who reported a history of diseases associated with depression, and population density of inhabited region.

^b^Data from the Statistics Bureau in Japan.

^c^Stroke, heart disease, diabetes mellitus, cancer, dementia, and Parkinson’s disease.

For women, overall, a similar tendency in those associations was observed, but the associations were weaker than those obtained for men. Within social participation, participation in sports clubs/groups was inversely associated with the SII (adjusted β = −0.42, *P* < 0.001), but it was strongly attenuated by covariate adjustment (Table 4). Among the variables on perceived community changes, perceiving increased poverty (adjusted β = 0.23, *P* = 0.06) and widening income inequality were somewhat strongly associated with inequality in depressive symptoms (adjusted β = 0.32, *P* = 0.01), whereas an increase in employment was inversely associated (adjusted β = −0.32, *P* = 0.02 for RII).

**Table 4.** Association between income-based health inequality indices (slope index of inequality [SII] and relative index of inequality [RII]) in depressive symptoms by 1 standard deviation unit increase in community characteristics measures among older Japanese women (*n* = 52,958): result of multiple regression

	SII				RII			
	
	Crude		Adjusted^a^		Crude		Adjusted^a^	
			
	β	*P*	β	*P*	β	*P*	β	*P*
**Socio-demographic factors**								
% Income <200 JPY	0.19	0.100			−0.04	0.720		
% Education <9 years	0.10	0.384			0.23^*^	0.044		
% Age ≥65 years^b^	0.26^*^	0.022			0.01	0.918		
% Living alone	0.33^*^	0.003			0.27^*^	0.017		
% Have comorbidities^c^	0.25^*^	0.027			0.19	0.103		
Population density, /km^2 b^	0.14	0.215			−0.07	0.548		
Gini coefficient	0.21	0.068	−0.02	0.903	0.09	0.420	−0.01	0.938
**Social participation**								
Volunteer group	−0.14	0.223	−0.11	0.435	−0.28^*^	0.014	−0.24	0.100
Sports club/group	−0.42^*^	<0.001	0.03	0.797	−0.18	0.119	−0.23	0.086
Leisure activity club	−0.30	0.007	0.10	0.484	−0.08	0.494	−0.08	0.590
Senior citizens’ club	0.00	0.987	−0.19	0.231	−0.14	0.229	0.09	0.618
Neighborhood association	−0.10	0.392	−0.08	0.584	−0.25^*^	0.026	−0.18	0.247
Cultural group	−0.10	0.381	0.08	0.464	0.14	0.215	0.14	0.267
Health-promoting activities	0.02	0.875	−0.06	0.621	−0.05	0.664	0.11	0.419
Activities entailing passing on experience to others	−0.21	0.069	0.03	0.801	−0.09	0.455	−0.07	0.527
Local events	−0.09	0.461	−0.18	0.202	−0.24^*^	0.035	−0.17	0.287
Supporting older people requiring protection	0.10	0.382	−0.18	0.257	−0.04	0.727	0.03	0.874
Supporting older people requiring long-term care	0.06	0.596	−0.22	0.118	−0.04	0.759	0.01	0.962
Supporting parents raising children	−0.08	0.516	0.09	0.467	−0.18	0.122	−0.06	0.652
Local beautification activities	−0.11	0.357	0.05	0.758	−0.21	0.062	−0.12	0.462
**Social relationships**								
Having friends	−0.10	0.396	−0.05	0.727	−0.18	0.124	0.09	0.571
Receiving emotional social support	−0.28^*^	0.012	0.01	0.960	−0.24^*^	0.037	−0.04	0.744
Providing emotional social support	−0.19	0.095	−0.06	0.645	−0.07	0.552	0.17	0.193
Receiving instrumental social support	−0.28^*^	0.015	−0.04	0.854	−0.25^*^	0.032	0.01	0.956
Providing instrumental social support	−0.18	0.114	0.09	0.555	−0.14	0.240	0.25	0.125
Trusting people in the area (very much or moderately)	−0.28^*^	0.014	0.24	0.102	−0.30^*^	0.008	−0.12	0.451
Practicing reciprocity as a norm (very much or moderately)	−0.24^*^	0.036	0.04	0.808	−0.34^*^	0.003	−0.31	0.083
Having a sense of attachment to the neighborhood (very much or moderately)	−0.15	0.194	0.18	0.120	−0.09	0.425	0.11	0.388
Co-operating with neighbors (very much or moderately)	0.08	0.511	−0.05	0.794	−0.14	0.232	0.13	0.561
**Perceived changes in the area**								
Revitalization of the local economy	0.06	0.627	−0.02	0.872	0.08	0.475	0.12	0.283
Depression of the local economy	0.23^*^	0.043	−0.01	0.950	−0.07	0.539	−0.11	0.425
Deterioration of security	0.08	0.506	−0.04	0.764	0.18	0.124	0.15	0.242
More newcomers	−0.24^*^	0.036	−0.13	0.380	−0.01	0.965	−0.16	0.320
Decline in local festivities	0.26^*^	0.020	−0.08	0.494	0.03	0.818	0.05	0.713
Increase in unemployment	0.03	0.796	0.09	0.510	−0.22	0.051	−0.34^*^	0.022
Increase in poverty	0.13	0.273	0.23	0.064	−0.07	0.538	−0.23	0.089
Improvement of administrative services	−0.25^*^	0.031	0.06	0.576	−0.09	0.440	−0.04	0.728
Deterioration of administrative services	0.07	0.522	−0.09	0.495	−0.09	0.419	0.06	0.676
Widening income inequality	0.06	0.594	0.32^*^	0.013	−0.08	0.509	−0.04	0.798
More local activities	−0.29^*^	0.012	−0.11	0.366	−0.24^*^	0.039	−0.23	0.079
Fewer local activities	0.29^*^	0.012	−0.01	0.953	0.01	0.906	0.07	0.638
**Built environment**								
Graffiti or garbage	0.15	0.193	−0.21	0.072	0.05	0.666	−0.16	0.218
Exercise environment	−0.15	0.189	0.15	0.412	0.03	0.816	−0.25	0.186
Hills or steps	−0.13	0.247	0.02	0.893	<−0.01	0.989	−0.09	0.514
Risk of traffic accidents	0.15	0.182	−0.11	0.340	0.23^*^	0.044	0.21	0.103
Fascinating views	−0.05	0.637	0.17	0.137	0.05	0.682	−0.05	0.679
Shops selling fresh foods	−0.07	0.529	0.31	0.102	0.15	0.196	−0.04	0.838
Dangerous places to walk in alone at night	−0.13	0.266	−0.15	0.152	−0.10	0.388	−0.05	0.682
Places to feel free to drop in	−0.05	0.692	0.04	0.738	−0.14	0.213	<0.01	0.987

All factors were adjusted for age.

^*^P < 0.05.

^a^Adjusted for the proportion of the population aged 65 years or more, the proportion of people who live alone, the proportion of people who reported a history of diseases associated with depression, and population density of inhabited region.

^b^Data from the Statistics Bureau in Japan.

^c^Stroke, heart disease, diabetes mellitus, cancer, dementia, and Parkinson’s disease.

For both genders, multiple adjusted regression did not show a clear association between the perceived built environment and inequality in depressive symptoms.

We confirmed that VIFs were less than 5 in all the regression models reported above. Sensitivity analysis with the alternative GDS cut-off also indicated similar associations (eTable 2).

## DISCUSSION

Our analysis found that a higher prevalence of participation in local activities was inversely associated with income-based inequalities in depressive symptoms for men at the community level, an association which was most prominent for sports clubs/groups. Similarly, more exchanges in social support were also associated with less inequality in depressive symptoms among men. Conversely, a perceived increase in unemployment, poverty, and economic inequalities was associated with large inequalities in depressive symptoms among men and women. We did not obtain clear evidence on the association between the built environment and inequalities in depressive symptoms in either gender.

The inverse association between rich social participation and social support and less inequality in depressive symptoms for men was intuitive. Social participation could provide more opportunities for social connections/networks, which provide social support.^26^^–^^28^ Extensive empirical evidence is available on the beneficial health effects of social participation,^29^ networks,^13^^,^^30^ and support.^31^ Evidence suggests that some contextual characteristics (eg, community social capital) may increase opportunities for enriching social relationships.^32^ A community in which there are plenty of local activities could provide more opportunities for maintaining mental health. Our findings suggest that these effects may be stronger for socially vulnerable people, resulting in less depression inequality. Alternatively, our measure of those social conditions may simply show compositional associations. That is, communities in which there were more people participating in local activities were less poor and depressed, resulting in narrower income gaps and, in turn, less income-based inequality in depressive symptoms. However, given the small association between income inequality and depression inequality, compositional associations would not fully explain these results.

The remarkable association between participation in sports groups/clubs and less inequality in depression among men is interesting, suggesting the importance of types of community groups. Using principal component analysis, Aida et al have categorized community activities into horizontal and vertical groups. The former includes groups relating to sports, hobbies, volunteering, and citizens’ and consumer associations, while the latter includes organizations for political matters and industrial, professional, religious, and local neighborhood associations.^33^ Using the JAGES data, Kanamori et al suggest that participation in sports clubs/groups may have stronger protective effects for long-term care than simply engaging in physical activities alone.^34^ Group-based sports activities may play a role, due to the social capital, which may in turn contribute to health. Taken together, our findings could mean that horizontal groups, such as sports groups, may be more beneficial for the mental health of socially vulnerable populations, compared to vertical groups, such as neighborhood associations.

Potential associations between more depression inequality and perceived community macroeconomic deterioration in terms of increasing unemployment, poverty, and income inequality are also reasonable. Stresses due to unemployment and poverty are known risks for the depressive state.^35^ Our findings may mirror the increase in mental illness due to economic hardships. Moreover, social participation is evidently less in areas where there are large income inequalities; this may provide an alternative explanation for the link between income inequalities and mental health.^36^

We did not find clear evidence on the association between the built environment and depression inequality. A recent systematic review concluded that there is insufficient evidence to enable clear recommendations of environmental interventions aiming to reduce depression, as the mechanisms of the association of depression with built environment have not been adequately studied.^37^

Although further studies are warranted, the public health implications of this ecological study is that the development of community social capital and an environment in which more social exchanges occur may contribute to not only overall depression prevalence, but may also address socioeconomic inequalities.^38^^–^^40^ For example, using instrumental variable analysis, Hikichi et al identified the association between participation in “community salon” activities—local activities providing social gathering opportunities, which were run by community volunteers—and improved functional ability among an older Japanese sample.^41^ Hirai et al found that poorer individuals were more likely to participate in those “salons,” suggesting that those interventions may help reduce income-based inequality in physical and mental health.^42^

We found potentially stronger associations among men compared to women. This might be because of larger differences in the depressive symptoms displayed by men. Alternatively, the impact of social interactions on depression may be weaker among men.^43^

There are five main limitations in our study. As we have discussed above, the results of this ecological study include both contextual and compositional associations.^44^ However, the present study was successful, as we have found several hypothetical factors associated with inequalities in depressive symptoms, which provide important perspectives for more detailed and sophisticated analyses in the future.^45^ Further studies should use a multilevel modeling approach and examine if these local community factors may be associated with individual incidence of the depressive state and if income levels modify the associations. Second, we have not incorporated lagged associations between the community environment and mental health.^46^ Future studies should use longitudinal data. Third, the random error of estimated inequality measures might be large in small communities, due to small sample sizes. Fourth, we repeated regression analyses with various independent variables, which increases the chances of type I error. Nonetheless, this is trivial, as the primary purpose of this study was to identify potential community factors associated with mental health inequality. Fifth, the PPV of GDS-15 using a cut-off score of 10 is low. Thus, there is a possibility of misclassification bias of depressive symptoms. However, a previous study has pointed out the low specificity of detecting depression using a lower cut-off point.^47^ Therefore, we adopted the cut-off of 9/10, following a previous Japanese study.^3^ Furthermore, since similar associations were found from sensitivity analyses using a cut-off of 4/5, the main results might not be so distorted.

### Conclusion

Future studies aiming to identify community-level factors contributing to the reduction of inequalities in mental illness should consider an environment promoting community participation and social relationships, as well as changes in community macroeconomic statuses. Tackling health inequalities has increasingly become an important public health activity worldwide.^48^^,^^49^ Further research is needed to discover triggers for reducing health inequalities in the depressive state among individuals with different income levels and to identify effective interventions.

## APPENDIX A. SUPPLEMENTARY DATA

eTable 1. Definitions of community-level factors investigated in this study

eTable 2. Result of sensitivity analysis using linear regression analysis: association between income-based health inequality indices (slope index of inequality [SII] and relative index of inequality [RII]) in depressive tendency by 1 standard deviation unit increase in community characteristics measures among Japanese older people
