# Supplementary material for: Community Social Capital, Built Environment, and Income-Based Inequality in Depressive Symptoms Among Older People in Japan: An Ecological Study From the JAGES Project
Source: J Epidemiol. 2018 Mar 5;28(3):108–16. doi: 10.2188/jea.JE20160216 (PMC5821687; doi:10.2188/jea.JE20160216)
Supplement: Supplementary file 1 [file je-28-108-s001.pdf]

eTable 1. Definitions of community-level factors investigated in this study

Factors	Description
**Socio-demographic factors**	Proportion of the following:
% Income <200 JPY	People with equivalized income, calculated by dividing the answer for "What was your pre-tax annual household income for 2012 (including pension)?" by the square root of the number of household members, less than 200 JPY
% Education <9 years	Percentage of people who responded "less than 6 years" and "6–9 years" to "How many years of formal education have you had?"
% Age ≥65 years	Population aged 65 years or more (%) [a]
% Living alone	People who are living alone (%)
% Have comorbidities	Proportion of people who reported past history of diseases associated with depression (stroke, heart disease, diabetes mellitus, cancer, dementia, and Parkinson's disease) (%)
Population density, /km^2	Population density by inhabited region(/km^2)[a]
**Social participation**	Sum of the percentage of people who responded, "4 or more times per week," "2–3 times per week," "once a week," "1–3 times per month," and "a few times per year" to "How often do you attend activities for the following groups?":
Volunteer group	Volunteer group
Sports club/group	Sports group or club
Leisure activity club	Leisure activity club
Senior citizens' club	Senior citizens' club
Neighborhood association	Neighborhood association or residents' association
Cultural group	Study or cultural group
Health-promoting activities	Nursing care prevention or health-building activities
Activities entailing passing on experience to others	Activities to teach skills or pass on experiences to others
Local events	Local events
Supporting older people requiring protection	Activities to support older people requiring protection
Supporting older people requiring long-term care	Activities to support older people requiring nursing care
Supporting parents raising children	Activities to support parents raising children
Local beautification activities	Activities entailing improvement (beautification) of local living arrangements
**Social relationships**	

Having friends	Sum of the percentage of people who responded "4 or more times per week," "2–3 times per week," "once a week," "1–3 times per month," and "a few times per year" to "How often do you see your friends?"
Receiving emotional social support	"Do you have someone who listens to your concerns and complaints?"
Providing emotional social support	"Do you listen to someone's concerns and complaints?"
Receiving instrumental social support	"Do you have someone who looks after you when you are sick and confined to a bed for a few days?"
Providing instrumental social support	"Do you look after someone when he/she is sick and confined to a bed for a few days?"
Trusting people in the area ("Very much" or "Moderately")	"Do you think that people living in your area can be trusted, in general?"
Practicing reciprocity as a norm ("Very much" or "Moderately")	"Do you think that people living in your area try to help others in most situations?"
Having a sense of attachment to the neighborhood ("Very much" or "Moderately")	"How attached are you to the area in which you live?"
Co-operating with neighbors	Percentage of people who responded, "Mutual consultation, lending and borrowing daily commodities, co-operation in daily life" to "What kind of interactions do you have with people in your neighborhood?"
**Perceived changes in the area**	"Which of the following changes have you seen in the area in which you live?"
Revitalization of the local economy	Revitalization of the local economy
Depression of the local economy	Depression of the local economy
Deterioration of security	Deterioration of security
More newcomers	More newcomers
Decline in local festivities	Decline of the local festival
Increase in unemployment	Increase in unemployment
Increase in poverty	Increase in poverty
Improvement of administrative services	Improvement of administrative services
Deterioration of administrative services	Deterioration of administrative services
Widening income inequality	Widening income inequality
More local activities	Increasing interaction or activities among community residents

Fewer local activities	Declining interaction or activities among community residents
**Built environment**	"Are the following present within walking distance of your home (within about 1 km)?":
Graffiti or garbage ("Many" or "Some")	Locations with noticeable graffiti or undisposed garbage
Exercise environment ("Many" or "Some")	Parks or foot paths suitable for exercise or walking in
Hills or steps ("Many" or "Some")	Locations difficult for walking, such as hills or steps
Risk of traffic accidents ("Many" or "Some")	Roads or crossroads with a great risk of traffic accidents
Fascinating views ("Many" or "Some")	Fascinating views or buildings
Shops selling fresh foods ("Many" or "Some")	Shops, facilities, or wagon retailers selling fresh foods (e.g., meat, fish, vegetables, and fruits)
Dangerous places to walk in alone at night ("Many" or "Some")	Dangerous places when walking alone at night
Places to feel free to drop in ("Many" or "Some")	Houses or facilities where you feel free to drop in

[a] Data from the Statistics Bureau in Japan

eTable 2. Result of sensitivity analysis using linear regression analysis: association between income-based health inequality indices (slope index of inequality [SII] and relative index of inequality [RII]) in depressive tendency by 1 standard deviation unit increase in community characteristics measures among Japanese older people

	Men								Women							
	SII				RII				SII				RII			
	Crude		Adjusted [a]		Crude		Adjusted [a]		Crude		Adjusted [a]		Crude		Adjusted [a]	
	β	P	β	P	β	P	β	P	β	P	β	P	β	P	β	P
**Socio-demographic factors**																
% Income <200 JPY	−0.07	0.566			−0.29 *	0.010			−0.17	0.149			−0.30 *	0.008		
% Education <9 years	0.11	0.342			−0.14	0.231			−0.05	0.675			−0.16	0.161		
% Age ≥65 years[b]	0.37 *	0.001			0.29 *	0.010			0.20	0.079			0.18	0.120		
% Living alone	0.02	0.884			−0.08	0.467			0.08	0.465			0.02	0.860		
% Have comorbidities[c]	0.25 *	0.032			0.32 *	0.005			0.21	0.068			0.28 *	0.016		
Population density, /km^{2}[b]	0.15	0.184			−0.01	0.945			0.07	0.558			−0.04	0.713		
**Social participation**																
Volunteer group	−0.16	0.166	−0.06	0.673	−0.08	0.479	0.12	0.420	0.02	0.831	0.12	0.440	0.02	0.837	0.15	0.316
Sports club/group	−0.13	0.261	−0.13	0.325	0.17	0.148	0.15	0.286	0.07	0.521	0.13	0.367	0.23 *	0.048	0.24	0.084
Leisure activity club	−0.13	0.253	−0.13	0.372	0.19	0.093	0.23	0.131	0.08	0.482	0.18	0.246	0.24 *	0.040	0.31 *	0.048
Senior citizens' club	−0.16	0.172	0.04	0.804	−0.20	0.088	0.11	0.531	−0.07	0.528	0.05	0.770	−0.12	0.314	0.08	0.645
Neighborhood association	−0.26 *	0.024	−0.20	0.190	−0.26 *	0.024	−0.10	0.507	−0.03	0.792	0.09	0.550	−0.09	0.416	0.05	0.752
Cultural group	<0.01	0.971	−0.06	0.644	0.29 *	0.012	0.22	0.077	0.16	0.153	0.16	0.195	0.35 *	0.002	0.33 *	0.007
Health-promoting activities	−0.10	0.408	0.04	0.776	−0.09	0.458	0.14	0.310	0.11	0.341	0.27	0.051	0.09	0.455	0.31 *	0.028
Activities entailing passing on experience to others	0.09	0.429	0.11	0.303	0.31 *	0.006	0.31 *	0.005	0.20	0.082	0.19	0.095	0.28 *	0.014	0.25 *	0.029
Local events	−0.22	0.054	−0.15	0.321	−0.18	0.111	−0.01	0.925	0.09	0.447	0.27	0.088	0.07	0.566	0.29	0.075
Supporting older people requiring protection	0.04	0.704	0.09	0.585	0.07	0.564	0.30	0.090	0.20	0.089	0.39 *	0.029	0.19	0.097	0.46 *	0.010
Supporting older people	0.04	0.728	0.05	0.754	0.05	0.641	0.16	0.305	0.23 *	0.048	0.35 *	0.023	0.23 *	0.042	0.41 *	0.008

requiring long-term care																
Supporting parents raising children	<0.01	0.968	0.21	0.114	0.03	0.789	0.33 *	0.017	−0.17	0.132	−0.17	0.231	−0.14	0.236	−0.05	0.721
Local beautification activities	-0.22	0.051	−0.18	0.265	−0.20	0.084	0.01	0.972	−0.04	0.723	0.07	0.683	−0.05	0.697	0.16	0.336
**Social relationships**																
Having friends	−0.34 *	0.002	−0.12	0.428	−0.17	0.140	0.15	0.353	−0.08	0.506	0.11	0.514	−0.07	0.542	0.10	0.536
Receiving emotional social support	−0.22	0.059	0.05	0.756	−0.17	0.139	0.19	0.251	−0.16	0.159	−0.05	0.768	−0.21	0.066	−0.09	0.577
Providing emotional social support	−0.45 *	<0.001	−0.31 *	0.018	−0.26 *	0.024	−0.08	0.583	−0.19	0.090	-0.09	0.505	−0.10	0.390	0.04	0.774
Receiving instrumental social support	−0.17	0.144	0.07	0.579	−0.05	0.683	0.12	0.344	−0.11	0.361	0.01	0.929	0.03	0.792	0.14	0.285
Providing instrumental social support	−0.47 *	<0.001	-0.53 *	0.011	−0.31 *	0.005	−0.15	0.495	−0.15	0.200	0.13	0.559	−0.10	0.403	0.29	0.186
Trusting people in the area (very much or moderately)	−0.19	0.095	0.21	0.190	−0.09	0.450	0.40 *	0.015	−0.22	0.051	−0.16	0.347	−0.14	0.240	0.05	0.762
Practicing reciprocity as a norm (very much or moderately)	−0.22	0.051	0.05	0.772	−0.16	0.165	0.21	0.233	−0.04	0.723	0.12	0.509	−0.06	0.599	0.11	0.551
Having a sense of attachment to the neighborhood (very much or moderately)	−0.29 *	0.010	−0.13	0.279	−0.16	0.162	−0.02	0.849	0.02	0.879	0.13	0.305	0.05	0.639	0.15	0.241
Co-operating with neighbors (very much or moderately)	−0.22	0.053	−0.19	0.078	−0.10	0.397	−0.07	0.562	0.14	0.221	0.21	0.068	0.11	0.352	0.17	0.134
**Perceived changes in the area**																

Revitalization of the local economy	0.09	0.429	0.11	0.432	−0.10	0.406	−0.04	0.772	0.15	0.200	0.16	0.253	−0.04	0.704	−0.04	0.758
Depression of the local economy	0.12	0.298	0.05	0.695	0.16	0.177	0.04	0.782	−0.01	0.957	<0.01	0.990	0.05	0.679	0.02	0.891
Deterioration of security	−0.08	0.485	−0.28	0.060	0.06	0.611	−0.18	0.253	0.04	0.739	0.09	0.556	0.16	0.163	0.21	0.190
More newcomers	0.11	0.336	0.12	0.333	0.05	0.657	0.12	0.371	0.15	0.206	0.12	0.347	0.06	0.609	0.05	0.688
Decline in the local festivities	0.08	0.464	0.18	0.219	−0.07	0.559	0.07	0.651	−0.08	0.464	−0.20	0.203	−0.20	0.088	−0.29	0.061
Increase in unemployment	0.15	0.183	0.05	0.727	−0.07	0.538	−0.13	0.350	−0.07	0.523	−0.22	0.107	−0.20	0.084	−0.31 *	0.023
Increase in poverty	−0.27 *	0.016	−0.22	0.055	−0.14	0.217	−0.13	0.252	−0.03	0.803	0.02	0.838	0.03	0.804	0.05	0.665
Improvement of administrative services	−0.11	0.340	0.03	0.852	−0.22	0.060	-0.03	0.841	−0.11	0.342	−0.04	0.781	−0.20	0.077	−0.11	0.482
Deterioration of administrative services	0.14	0.222	0.31 *	0.024	−0.03	0.823	0.19	0.175	−0.12	0.300	−0.13	0.369	−0.20	0.078	−0.15	0.297
Widening income inequality	−0.27 *	0.016	-0.23	0.063	−0.11	0.344	−0.15	0.252	0.12	0.316	0.20	0.139	0.16	0.154	0.18	0.186
More local activities	0.05	0.644	0.07	0.653	−0.01	0.915	0.11	0.463	0.15	0.190	0.22	0.152	0.02	0.862	0.08	0.589
Fewer local activities	−0.10	0.390	0.27	0.228	−0.20	0.074	0.14	0.550	<0.01	0.991	0.26	0.262	−0.08	0.476	0.16	0.480
**Built environment**																
Graffiti or garbage	0.23	0.042	0.06	0.636	0.12	0.279	−0.06	0.628	0.16	0.163	0.13	0.345	0.12	0.290	0.07	0.597
Exercise environment	0.06	0.593	-0.11	0.572	0.23 *	0.046	0.08	0.685	0.03	0.785	−0.04	0.833	0.13	0.273	0.01	0.967
Hills or steps	0.15	0.202	0.13	0.324	0.25 *	0.030	0.21	0.119	0.18	0.113	0.10	0.481	0.32 *	0.005	0.24	0.073
Risk of traffic accidents	0.16	0.157	0.14	0.257	0.10	0.409	<0.01	0.972	−0.01	0.906	−0.01	0.930	−0.02	0.860	−0.05	0.684
Fascinating views	0.12	0.287	0.01	0.958	0.25 *	0.026	0.13	0.277	0.17	0.148	0.12	0.332	0.23 *	0.043	0.17	0.192
Shops selling fresh foods	0.10	0.374	−0.19	0.345	0.21	0.065	−0.11	0.601	−0.07	0.548	-0.54 *	0.010	0.01	0.942	−0.50 *	0.016
Dangerous places to walk in alone at night	−0.08	0.469	-0.01	0.962	−0.05	0.683	−0.02	0.875	0.03	0.805	0.09	0.444	0.13	0.269	0.17	0.152
Places to feel free to drop in	−0.20	0.088	−0.07	0.562	−0.20	0.078	−0.06	0.624	−0.03	0.819	0.07	0.618	−0.11	0.348	−0.02	0.862

All factors were adjusted for age.

* $P<0.05$

[a] Adjusted for the proportion of the population aged 65 years or more, the proportion of people who live alone, the proportion of people who reported a history of diseases associated with depression, and population density of inhabited regions

[b] Data from the Statistics Bureau in Japan

[c] Stroke, heart disease, diabetes mellitus, cancer, dementia, and Parkinson’s disease
